# Vitamin D insufficiency is associated with inflammation and deregulation of adipokines in patients with metabolic syndrome

**DOI:** 10.1186/s12902-022-01141-0

**Published:** 2022-09-07

**Authors:** Zeinab Khademi, Soudabeh Hamedi-Shahraki, Farshad Amirkhizi

**Affiliations:** 1 Department of Public Health, Sirjan School of Medical Sciences, Sirjan, Iran; 2 Department of Epidemiology and Biostatistics, Faculty of Public Health, Zabol University of Medical Sciences, Zabol, Iran; 3 Department of Nutrition, Faculty of Public Health, Zabol University of Medical Sciences, Bagheri St., Shahid Rajaei St., 9861615881, Zabol, Iran

**Keywords:** Metabolic syndrome, Vitamin D, Inflammation, Adipokines, Visfatin, Resistin

## Abstract

**Background:**

Previous studies have been reported that vitamin D deficiency increased the risk of metabolic syndrome (MetS). Nonetheless, the exact mechanisms underlying this association is unclear. Besides, inflammation and deregulation of adipokines secretion have been recognized as pivotal factors that contribute to the pathogenesis of these conditions. Therefore, we assessed whether serum vitamin D status is associated with serum levels of adipokines and inflammatory markers in these patients.

**Methods:**

This case-control study was carried out among 65 patients with MetS who had vitamin D insufficiency (cases) and 130 MetS patients who had vitamin D sufficiency (controls). Cases and controls were recruited from among those referred to health centers in Zabol County, Iran. Vitamin D insufficiency was regarded as a serum 25-hydroxyvitamin D [25(OH)D] concentration below 30 ng/ml. Serum concentrations of leptin, adiponectin, visfatin, and resistin and also adiponectin/leptin ratio along with serum levels of interleukin 6 (IL-6), IL-10 and tumor necrosis factor-alpha (TNF-α), were evaluated.

**Results:**

Serum levels of leptin, resistin, and TNF-α were significantly higher, whereas, serum adiponectin and adiponectin/leptin ratio were significantly lower in cases than the controls. There was no significant difference in serum visfatin, IL-6, and IL-10 between the groups. Serum levels of 25(OH)D were inversely correlated with leptin, resistin, and TNF-α in both unadjusted models and after adjustment for potential confounders.

**Conclusion:**

Our findings indicated that vitamin D insufficiency in MetS patients is associated with increased inflammation and serum adipokine abnormalities which may be associated with developing metabolic complications in these patients.

## Introduction

Vitamin D deficiency is considered one of the commonest disorders in the world [1]. In Iran, a Middle Eastern country, recent investigations showed that more than half of the population suffers from vitamin D deficiency [2].

Previous evidence focused on the effect of vitamin D on maintaining bone health and calcium homeostasis [3]. Vitamin D deficiency is linked to the high risk of several chronic and autoimmune disorders including cardiovascular diseases, metabolic syndrome (MetS), diabetes, and multiple sclerosis [4, 5]. Although the exact mechanism by which vitamin D deficiency contributes to the pathogenesis of these conditions is not fully understood. One possible explanation can be the anti-inflammatory properties of vitamin D [6], considering the role of inflammation in the pathology of several chronic conditions [7]. Vitamin D influences the generation and function of adipokines and therefore inflammatory reaction in adipose tissue [1, 8]. Several investigations have assessed the association between vitamin D and metabolic syndrome [9, 10]. Evidence indicates that in the US, the prevalence of MetS increased as the serum vitamin D decreased [11]. The link between this vitamin and adipokines and inflammatory factors, especially in MetS patients, received less attention. Pott-Junior et al. [12] found that when comparing metabolic syndrome subjects, serum levels of interleukin 10 (IL-10), IL-1α, and tumor necrosis factor-alpha (TNF-α) showed a trend towards higher levels in subjects with vitamin D deficiency. In intervention studies, vitamin D supplementation can increase adipokine concentrations in obese or overweight adults [8]. Considering limited data available on this subject, especially in developing countries, we examined vitamin D status in relation to adipokines and inflammatory markers in patients with metabolic syndrome in a case-control study.

## Materials and methods

### Samples

A case-control study was conducted on subjects aged 20–50 years between April 2021 and January 2022 in Zabol County, northeast Iran. Cases were patients diagnosed with MetS based on the international criteria, including meeting a minimum of three of the following criteria: triglyceride (TG) ≥ 150 mg/dl, fasting serum glucose (FSG) ≥ 100 mg/dl, blood pressure (BP) ≥ 130/ ≥ 85 mmHg, waist circumference (WC) ≥ 95 cm (both males and females) and high density lipoprotein cholesterol (HDL-c) < 40 mg/dl (males) or < 50 mg/dl (females), [13] who had serum 25-hydroxyvitamin D [25(OH)D] smaller than 30 ng/ml (vitamin D insufficiency). The Iranian National Committee of Obesity’s report introduced the cutoff value for WC for abdominal obesity for the Iranian population [14]. Cases were recruited from among those referred to health centers affiliated with Zabol University of Medical Sciences. Every health center offers primary health care for families within its coverage or reach. Serum adiponectin levels as a key variable were obtained from Rambhojan et al. study [15], was used to estimate the sample size. Considering the study power of 80%, a type I error of 5%, and the ratio of controls to cases as 2, we required 65 cases and 130 controls for this study.

Patients with MetS who had a prior history of cardiovascular diseases, liver dysfunction, renal failure, hypo or hyperthyroidism, inflammatory disease, cancer, and cases who had a history of hormone replacement therapy were not included in this project. The patients were also excluded if taking omega-3 or antioxidant supplements, such as vitamin C, vitamin E, selenium, and also lipid-lowering and antihypertensive agents at baseline or within 3 months prior to the interview.

Controls were patients with MetS and serum 25(OH)D ≥ 30 ng/ml (vitamin D sufficiency) and were selected from subjects visiting the same health centers. Control subjects were matched with cases regarding sex, age (±2 years), and BMI (±1 kg/m^2^). The exclusion criteria for controls included the presence of any inflammatory disease, cardiovascular diseases, cancer, liver diseases, thyroid disorders, and kidney dysfunctions, being pregnant or lactating, and adherence to special diets. In addition, we did not include patients with a history of any hormone replacement therapy. Similarly, those who took omega-3 or other antioxidant supplements, such as vitamin C, vitamin E, and selenium, and also lipid-lowering and antihypertensive agents at baseline or within 3 months before the interview were not qualified. Eligible subjects, including 65 cases and 130 controls were recruited for the study.

All subjects signed an informed consent after explaining the aims and the study methodology. The Ethics Committee of Zabol University of Medical Sciences approved the research (IR.ZBMU.REC.1399.156).

### Demographic characteristics, anthropometric, and body composition assessments

At baseline, demographic characteristics, such as age, sex, smoking status, educational condition, and medical history were obtained for each patient using a self-administered demographic and medical questionnaire. Body weight and height were assessed with minimal clothes without shoes by the Seca scale (Germany) with the accuracy of 100 g and 0.5 cm, respectively. The WC was assessed between the iliac crest and the lower rib margin following a normal expiration. BMI was calculated by dividing weight (kilograms) by height in square meters.

The visceral fat level (%VF) and body fat mass (%FM) percentage was measured by the bioelectrical impedance analysis (BIA) system (InBody S10, JMW140, Korea). To increase accuracy, the subjects were asked to prevent from intense or moderate exercises 1–2 h before the use of BIA and to urinate prior to tests. The same trained technician performed all measurements to reduce subjective error.

Blood pressure was evaluated in a sitting position following resting for 15 min in a quiet environment by a mercury sphygmomanometer. The average diastolic blood pressure (DBP) and systolic blood pressure (SBP) were noted by two readings with intervals of 5 min.

Habitual physical activity levels (PAL) within the past 7 days were evaluated according to the information achieved from the short form of the International Physical Activity Questionnaire (IPAQ) [16]. IPAQ has seven questions assessing the duration and frequency of individuals in “vigorous”, “moderate”, and “walking” activities, and also the time elapsed sitting during the last week. PA data were converted into energy expenditure estimates as metabolic equivalents (METs) by the use of published values [17]. For converting the IPAQ data into the physical activity during the week (MET-h/week), the number of hours elapsed in each category was multiplied by the certain MET score for the considered activity [18].

### Biochemical measurements

Following 10–12 hours of overnight fasting, a 10 ml venous blood sample was collected from all participants and centrifuged at 3500 rpm for 10 min for separating the sera. Fasting serum concentrations of glucose (FSG), HDL-c, and TG were evaluated by the standard enzymatic-colorimetric technique on an automatic biochemical Hitachi 717 analyzer (Hitachi Ltd., Tokyo, Japan) by commercial kits (Pars-Azmoon, Iran) on the blood sampling day. Then, the remnants of sera were stored at − 70 °C until assay time. Serum insulin levels were measured based on the radioimmunoassay method using the commercial kit (Immunotech, Prague, Czech Republic). Insulin resistance was estimated with the homeostasis model assessment method (HOMA-IR) using the suggested equation:  $\mathrm{HOMA}-\mathrm{IR}=\left[\mathrm{fasting}\;\mathrm{insulin}\;\left(\mathrm U/\mathrm l\right)\times\mathrm{fasting}\;\mathrm{glucose}\;\left(\mathrm{mg}/\mathrm{dl}\right)\right]/405$ [19].

Serum 25(OH)D levels were determined through enzyme-linked immunosorbent assay (ELISA) kits (DIAsource Immunoassays SA, Belgium) as instructed. Vitamin D sufficiency was regarded as a serum 25(OH)D level of 30 ng/ml or greater and those who had serum 25(OH)D levels below 30 ng/ml were considered insufficient based on the previous reported and Endocrine Society clinical practice instructions [20, 21].

To measure the serum concentrations of leptin, adiponectin, resistin, and visfatin, ELISA kits (Bioassay Technology Laboratory, Shanghai, China) were employed. Serum concentrations of IL-6 and IL-10 were determined using the Human ELISA kits (IBL-Immuno-Biological Laboratories Co, USA) based on the manufacturer’s instructions. Serum TNF-α levels were also measured using an ELISA kit (Beckman Coulter Immunotech, France). The intra- and inter-assay coefficients of variation (CVs) for all the biochemical assessments were from 6 to 10%, respectively.

### Statistical analysis

Data are presented as mean ± SD for quantitative data that had a normal distribution and frequency (percent) for qualitative data. The data with normal distribution are expressed as the median and interquartile range (IQR). The normality of the data distribution was checked by Kolmogorov–Smirnov test and a Q–Q plot. Comparison of demographic, anthropometric, and metabolic characteristics between controls and cases were done using the independent samples t-test and the chi-square test for continuous and categorical data, respectively. Also, between-group differences in non-normally and normally distributed adipokines and inflammatory markers were explored by independent samples t-test, ANOVA, and non-parametric Mann Whitney U test, where appropriate. To explore the relationship between serum concentrations of 25(OH)D with inflammatory markers and adipokines, multiple linear regression in crude (model 1) and adjusted model (model 2) were used. In the model 2, we adjusted for sex, age, smoking, BMI, physical activity level, and FSG. Analyses were done by SPSS 18 software. *P*-values smaller than 0.05 were regarded significant.

## Results

A total of 195 participants (65 cases and 130 controls) were assessed, including 66 (33.8%) males and 129 (66.2%) females. The mean (± SD) age and BMI of the participants were respectively 37.7 ± 5.6 years and 31.8 ± 2.0 kg/m^2^. The mean serum 25(OH)D concentrations in the participants were 34.1 ± 13.3 ng/ml.

Table 1 indicates the anthropometric, demographic, and metabolic characteristics of the participants. No significant difference was observed in the mean age, BMI, weight, WC, visceral fat, fat mass, serum insulin levels and physical activity values and also the distribution of participants regarding sex and smoking between cases and controls. Meanwhile, noticeable differences were found in the FSG (*P* = 0.004), HOMA-IR (*P* = 0.023) and serum levels of 25(OH)D (*P* < 0.001) between cases and controls.

**Table 1:** Demographic, anthropometric, and metabolic characteristics of cases and controls^a^

Variables	Patients with metabolic syndrome		*P*-value^b^
	vitamin D sufficient (Controls, *n* = 130)	vitamin D insufficient (Cases, *n* = 65)	
Male, *n* (%)	45 (34.6)	21 (32.3)	0.748
Age (years)	37.5 ± 5.6	38.0 ± 5.5	0.556
Weight (kg)	90.6 ± 12.1	89.7 ± 11.6	0.673
BMI (kg/m^2^)	31.6 ± 1.9	32.0 ± 2.1	0.289
WC (cm)	94.6 ± 9.4	95.4 ± 8.5	0.593
Fat mass (%)	42.8 ± 7.3	44.3 ± 7.3	0.185
Visceral fat (%)	12.8 ± 4.0	13.2 ± 4.1	0.534
PAL (MET-h/week)	30.5 ± 6.8	29.2 ± 6.6	0.221
Smokers, *n* (%)	31 (23.8)	14 (21.5)	0.718
FSG (mg/dl)	101.3 ± 8.8	106.1 ± 13.6	**0.004**
Insulin (μU/mL)	16.8 ± 5.2	18.1 ± 5.8	0.116
HOMA-IR	4.2 ± 1.5	4.8 ± 2.1	**0.032**
HDL-c (mg/dl)	46.5 ± 6.6	47.4 ± 6.3	0.412
TG (mg/dl)	191.5 ± 17.4	193.8 ± 22.9	0.453
SBP (mmHg)	126.5 ± 4.1	127.2 ± 3.8	0.304
DBP (mmHg)	82.9 ± 2.7	83.7 ± 3.2	0.083
25(OH)D (ng/ml)	41.2 ± 9.7	19.8 ± 6.2	**< 0.001**

*BMI* body mass index, *WC* waist circumference, *PAL* physical activity level, *MET* metabolic equivalent of task, *FSG* fasting serum glucose, *HOMA-IR* homeostasis model assessment of insulin resistance, *HDL-C* high-density lipoprotein cholesterol, *TG* triglyceride, *SBP* systolic blood pressure, *DBP* diastolic blood pressure

*P*-values of statistical significance (*P* < 0.05) are indicated in bold

^a^Data are shown as mean ± standard deviation for continuous variables and number (%) for categorical variables

^b^Resulted from independent samples *t*-test or Pearson chi-square test for continuous and categorical variables, respectively

The comparisons of the serum levels of adipokines and inflammatory markers between the study groups are presented in Table 2. The mean serum concentrations of leptin (*P* = 0.002), resistin (*P* = 0.037), and TNF-α (*P* < 0.001) were significantly higher in the cases with vitamin D insufficiency (cases) than those with vitamin D sufficiency (controls). In addition, patients with vitamin D sufficiency also had higher serum levels of adiponectin (*P* = 0.003) and leptin / adiponectin ratio (*P* < 0.001) than patients with vitamin D insufficiency.

**Table 2:** Serum concentrations of adipokines and inflammatory markers in the participants by the vitamin D status^a^

Variables	Patients with metabolic syndrome		*P*-value
	vitamin D sufficient (Controls, *n* = 130)	vitamin D insufficient (Cases, *n* = 65)	
Leptin (ng/ml)	22.9 (18.4, 40.7)	32.6 (22.5, 56.2)	**0.002**^**b**^
Adiponectin (μg/ml)	16.3 (12.3, 25.8)	12.9 (11.1, 17.3)	**0.003**^**b**^
Adiponectin/Leptin ratio	0.72 (0.43, 0.97)	0.48 (0.27, 0.66)	**< 0.001**^**b**^
Visfatin (ng/ml)	16.2 ± 11.2	15.0 ± 8.4	0.587^c^
Resistin (ng/ml)	5.79 ± 1.01	6.09 ± 0.77	**0.037**^**c**^
TNF-α (pg/ml)	24.7 ± 4.7	27.3 ± 4.5	**< 0.001**^**c**^
IL-6 (pg/ml)	5.00 (4.39, 6.42)	5.28 (4.51, 6.10)	0.539^b^
IL-10 (pg/ml)	36.4 (30.7, 43.8)	34.7 (29.7, 45.1)	0.518^b^

*TNF-α* tumor necrosis factor-α, *IL-6* interleukin-6, *IL-10* interleukin-10

*P*-values of statistical significance (*P* < 0.05) are indicated in bold

^a^Data are shown as mean ± standard deviation for variables with normal distribution and as median (IQR) for variables with skewed distribution (i.e.*,* leptin, adiponectin, adiponectin/leptin ratio, IL-6, and IL-10)

^b^Resulted from Mann–Whitney *U* test

^c^Resulted from Independent samples *t*-test

The models’ multiple linear regression results, which investigated the associations between serum 25(OH)D as the independent variable and serum adipokines and inflammatory markers as dependent variables are denoted in Table 3. Multiple linear regression analyses in all patients confirmed the vitamin D dependency of serum leptin, adiponectin, resistin, and TNF-α as well as adiponectin/leptin ratio. The serum 25(OH)D concentrations showed an inverse correlation with serum concentrations of leptin (*β* = − 0.254, *P* < 0.001), resistin (*β* = − 0.181, *P* = 0.011) and TNF-α (*β* = − 0.252, *P* < 0.001) and positively correlated with serum levels of adiponectin (*β* = 0.387, *P* < 0.001) and adiponectin/leptin ratio (*β* = 0.423, *P* < 0.001) in an unadjusted model (model 1). In addition, there were significant inverse correlations between serum 25(OH)D levels with FSG (*β* = − 0.189, *P* = 0.007) and HOMA-IR (*β* = − 0.206, *P* = 0.004) (data not shown). The aforementioned associations were significant after adjustment for potential confounders, such as age, physical activity level, sex, smoking, BMI, and FSG (model 2).

**Table 3:** Multiple linear regression analysis for the association between serum 25(OH)D concentrations with adipokines and inflammatory markers (*n* = 195)

Model	B	S.E.	β	*P*-value
Leptin (ng/ml)				
Model 1^a^	− 0.442	0.121	− 0.254	**< 0.001**
Model 2^b^	− 0.384	0.119	−0.223	**0.003**
Adiponectin (μg/ml)				
Model 1	0.423	0.073	0.387	**< 0.001**
Model 2	0.432	0.071	0.391	**< 0.001**
Adiponectin/Leptin ratio				
Model 1	0.021	0.003	0.423	**< 0.001**
Model 2	0.019	0.003	0.432	**< 0.001**
Visfatin (ng/ml)				
Model 1	0.041	0.054	0.055	0.450
Model 2	0.034	0.061	0.041	0.487
Resistin (ng/ml)				
Model 1	−0.013	0.005	−0.181	**0.011**
Model 2	−0.013	0.004	−0.181	**0.013**
TNF-α (pg/ml)				
Model 1	−0.091	0.025	−0.252	**< 0.001**
Model 2	−0.091	0.022	−0.243	**< 0.001**
IL-6 (pg/ml)				
Model 1	−0.011	0.021	−0.039	0.592
Model 2	−0.013	0.211	−0.051	0.340
IL-10 (pg/ml)				
Model 1	0.030	0.051	0.042	0.563
Model 2	0.029	0.041	0.039	0.461

*TNF-α* tumor necrosis factor-α, *IL-6* interleukin-6, *IL-10* interleukin-10, *B* unstandardized coefficient, *S.E.* standard error

*P*-values of statistical significance (*P* < 0.05) are indicated in bold

^a^Model 1: unadjusted model

^b^ Model 2: adjusted for age, sex, smoking, physical activity level, BMI, and FSG

## Discussion

There was a significant inverse association between serum 25(OH)D and leptin, resistin, and TNF-α. Moreover, a positive link between serum 25(OH)D and adiponectin and adiponectin/leptin ratio was observed, which remained significant after adjustment for several potential confounders. As far as we know, this is among the first studies on this relationship.

Metabolic syndrome is a complex cluster of several conditions including dyslipidemia, elevated blood pressure, impaired glucose homeostasis, and abdominal obesity [22, 23]. The relationship between vitamin D deficiency, metabolic syndrome, and its components has been assessed before. Several observational studies found that vitamin D deficiency is associated with metabolic syndromes [6, 24–26]. We focused on the correlation between vitamin D serum concentrations and adipokines and inflammatory biomarkers in those suffering from MetS. A significant inverse relationship was detected between serum 25(OH)D concentrations and TNF-α. However, we failed to find any significant link between serum 25(OH)D concentrations and IL-10 and IL-6. Similar to our findings, Pott-Junior et al. [12] found the same inverse link between vitamin D and TNF-α, when comparing them within metabolic syndrome subjects. Furthermore, these researchers observed that serum levels of IL-10 trend towards higher levels in subjects with vitamin D deficiency. Moreover, findings from two population-based studies indicated an inverse link between serum 25(OH)D and IL-6 [27, 28]. In our study, a significant inverse association was found between serum 25(OH)D with resistin and leptin . Moreover, we observed a positive link between serum 25(OH)D with adiponectin and adiponectin/leptin ratio. In a cross-sectional study assessing non-alcoholic fatty liver disease (NAFLD) patients, Chen et al. [29] found no association between vitamin D and TNF-α or adiponectin levels. In a study on the Thai population, there was a relationship between insufficient vitamin D status and lower serum adiponectin in cases who had abnormal glucose tolerance [30]. In another study on diabetes patients, an inverse correlation between 25(OH)D and serum resistin was revealed. While investigators failed to find any link between this vitamin and other adipokines including leptin and adiponectin [31].

The exact mechanisms by which vitamin D deficiency contributes to adipokines and inflammatory biomarkers levels have not been fully discovered yet. Although the vitamin D receptor (VDR) in adipose tissue led to the assumption of a direct role of vitamin D in regulating adipokines [32]. One possible mechanism might be downregulation of the TNF-α gene, known to affect adiponectin synthesis, by vitamin D [33]. Vitamin D also downregulates the renin-angiotensin system in adipose tissue and higher angiotensin concentrations seem to increase dysfunctional adipocytes and reduce adiponectin generation [34]. Regarding inflammatory cytokines, evidence shows that vitamin D can reduce inflammatory cytokines and therefore a reduction in systemic inflammation through binding to VRD receptors [35]. In fact, evidence showed that secretion of nuclear factor κB (NFκB) and release of IL-6 are reduced by vitamin D [6]. In animal studies, vitamin D3-deficient mice displayed higher gene transcript levels of inflammatory mediators, such as TNF-α, IL-1, and IL-6 [36].

This study has several strengths as well as limitations. Being among the first studies examining the association between vitamin D with serum adipokines and inflammatory markers in metabolic syndrome patients and adjustment for several potential confounders are among our strengths. However, some limitations must be mentioned. First, the case-control design of the present study, which prohibits us from concluding causality. Secondly, the effect of some residual confounders which cannot be ignored. Finally, we could not measure serum C-reactive protein (CRP) concentrations that is prototypic marker of inflammation.

## Conclusions

In conclusion, the findings of this study indicate that in patients with MetS, serum levels of leptin, resistin, and TNF-α inversely associated with serum vitamin D status. In addition, a positive link between this vitamin and adiponectin and adiponectin/leptin ratio was observed. Indeed, these findings confirm favorable role of vitamin D in the improvement of inflammatory and metabolic markers in patients with MetS. Further large sample investigations are needed to validate our findings.

## Data Availability

The datasets used and/or analyzed during the current study available from the corresponding author on reasonable request.

## Abbreviations

MetS: Metabolic syndrome
TNF-α: Tumor necrosis factor alpha
IL-6: interleukin 6
IL-10: interleukin 10
BMI: Body mass index
WC: Waist circumference
